# Prediction of Welding Deformation Using the Thermal Elastic–Plastic Finite Element Method by Considering Welding Interpass Temperature

**DOI:** 10.3390/ma17153656

**Published:** 2024-07-24

**Authors:** Young-Hwan Han, Hun-Bong Lim, Tae-Sung Shin, Hyun-Ik Yang

**Affiliations:** 1 Department of Mechanical Design Engineering, Hanyang University, 222 Wangsimni-ro, Seongdong-gu, Seoul 04763, Republic of Korea; hyh1994@hanyang.ac.kr; 2 Department of Mechanical Design Engineering, Myongji College, 134 Gajwa-ro, Seodaemun-gu, Seoul 03656, Republic of Korea; 3 Textile Innovation R&D Department, Korea Institute of Industrial Technology (KITECH), 143 Hanggaul-ro, Sangnok-gu, Ansan-si 15588, Republic of Korea

**Keywords:** finite element method, multi-pass welding, SUS304, thermal elastic–plastic finite element method, welding deformation

## Abstract

In this study, we propose a method for predicting welding deformation caused by multi-pass welding using the thermal elastic–plastic finite element method (TEP-FEM) by considering the interpass temperature. This method increases the interpass temperature, which has not been considered in the existing TEP-FEM, from 200 °C to 1000 °C, and simultaneously performs thermal and mechanical analyses. In addition, this method can also evaluate temperature history and the time it takes to weld. By predicting the welding deformation using this method, angular distortion prediction was reduced from 16.75 mm to 10.9 mm compared to the case where the interpass temperature was cooled to room temperature. Additionally, the deformation error was significantly reduced from 6.14% to 2.92% compared to that of the strain as directed boundary method used in a previous study. Additionally, our research demonstrated that interpass temperatures above 800 °C can result in increased deformation errors. In conclusion, it is essential to select an appropriate temperature to minimize deformation error.

## 1. Introduction

Welding is widely used in the mechanical industry. It is especially important in areas such as the ship-building industry and marine plant businesses because large structures are manufactured by welding of small structures. Welding is considered the most economical method for joining parts in terms of material utilization and manufacturing costs [1]. However, welding generates heat and stress, which can lead to unpreventable deformations, such as longitudinal shrinkage and angular distortion in structures. These deformations persist even after welding and cause errors in the structural assembly process [2]. Therefore, predicting welding deformation is very important for producing large structures. The most effective approach for predicting welding deformations is to gather information by conducting multiple welding operations. However, this approach is not economically viable for large structures. Thus, by using the finite element method (FEM), welding deformation can be predicted in advance without welding, regardless of the size of the structure.

This FEM simulation can be largely divided into thermal elastic–plastic FEM (TEP-FEM) and the simplified analysis method based on the inherent strain theory. In 1971, Ueda and Yamakawa [3] conducted welding analyses using TEP-FEM. This study was the first to utilize TEP-FEM and consider the temperature-dependent properties of a metal, including its elastic modulus, yield stress, and thermal expansion coefficient. Moreover, the authors evaluated the thermal history during welding and analyzed the transient thermal stresses. Since then, TEP-FEA has been studied and applied to various welding processes such as arc welding, laser welding, and electron beam welding. Ueda and Yuan [4] presented a method for predicting the intrinsic strain distribution and residual stress that occur during butt welding. Xia et al. [5] investigated the effects of the initial gap on the inherent strain and welding deformation in laser arc welding using FEM. Abbasi et al. [6] examined the temperature distribution during friction stir welding and assessed its influence on strain and heat generation. Hwang et al. [7] predicted the residual stress distribution of an 80mm thick plate joint during the EGW process. Wu and Kim [8] simulated the welding process of a thin plate and computed the inherent strain using TEP-FEM. They conducted an experiment to verify the accuracy of the proposed numerical model. Tian and Luo [9] quantitatively investigated the effects of process parameters (including welding current, voltage, and speed) and plate thickness on the in-plane inherent deformations in typical fillet welded joints.

Simplified analysis methods include equivalent load, equivalent strain, and strain as directed boundary (SDB) methods. Murakawa et al. [10] examined the deformations that occurred during welding by applying the equivalent load method. The authors also proposed the use of a gap element to improve the accuracy of the results. Kim et al. [11] used the equivalent strain method to facilitate the rapid and precise prediction of weld deformation by considering the residual stress of a curved double-bottom block, which is commonly used in shipbuilding. Lee and Chung [12] developed a modified equivalent load method by improving the existing method, which could not accurately estimate longitudinal bending. Additionally, the shell element-based elastic finite element (FE) approach was investigated, and the analysis of welding deformation was improved. Ha [13] described the SDB method, which expresses the internal shrinkage in welding areas as an arbitrary thermal expansion coefficient and temperature boundary condition. Ha [14] suggested a technique for determining the temperature boundary conditions for each type of welding joint and employed the SDB method to forecast the weld deformation of the unit specimen and block model.

However, as the size of welded structures has increased, welding and analysis simulations are no longer performed in a single pass. Consequently, numerous studies have been conducted to analyze the properties and performance of multi-pass welding. These studies focused on a wide range of factors, including weld quality, weld strength, welding speed, and welding efficiency. Ha et al. [15,16] conducted follow-up SDB studies to propose a method for determining the temperature boundary conditions for multi-pass welding. Vemanaboina et al. [17] performed simulations on the multi-pass gas tungsten arc welding of SS316L and studied its effects on heat and residual stress. Wu et al. [18] conducted a study predicting reserved clearance occurring in multi-pass double-sided arc welding. They also used the dynamic heat distribution model and TEP-FEM to calculate welding deformation and reduce errors in the experimental results.

However, most multi-pass welding studies have not considered the interpass temperature. The term “interpass temperature” refers to the temperature of the prior bead when welding is completed, and another bead is added to the top. The interpass temperature had a negative impact on the weld heat-affected zone (HAZ) and hydrogen cracking. When the interpass temperature increases and the size of the HAZ increases, the formation of martensite increases in the weld zone, reducing toughness and ductility. Therefore, in previous studies, the principle was to cool the welded part to room temperature during welding to maximize hydrogen release, prevent cracking of residual hydrogen, and minimize the size of the HAZ. However, as the interpass temperature increases, there are also advantages, such as reduced welding deformation and faster working time. In fact, at the sites where welding was performed, welding was conducted without allowing the weld zone to cool to room temperature to minimize the overall duration of the welding process, according to the instructions of a skilled welder. Recently, Han [19] predicted multi-pass welding deformation using the SDB method considering the interpass temperature, and reduced the maximum error from 56.2% to 6.14%. 

The SDB method can only confirm the final deformation results and thermal load and cannot confirm the temperature history that appears during welding. Therefore, in this study, we investigated the effect of the interpass temperature on HAZ by verifying the entire temperature history using TEP-FEM. Additionally, we predicted the deformation of multi-pass welds and confirmed the interpass temperature with the lowest welding deformation error.

## 2. Formulation of TEP-FEM

TEP-FEM was used to simulate the transient behavior of weld deformation and stress under time-based temperature loading. The fundamental principle of FEM is to view a structure as a collection of structural components that are interconnected at a limited number of nodal points where the equilibrium and compatibility criteria are fulfilled.

### 2.1. Basic Equations for Analysis of TEP-FEM

During the entire welding process, where fusion and solidification occur, the initial strain is a function of temperature, i.e., the thermal strain, and the increase in the stress of the element can be expressed as Equation (1).

(1) d σ=Ddε−C dT

where dσ represents the stress increment, D is the stiffness matrix, dε is the strain increment, C is the temperature matrix, and dT represents the temperature increment. The relationship between the increments in the nodal forces (dF) and nodal displacements (du) was established by applying the principle of virtual displacement, as shown in Equation (2).

(2) $$dF=\int B^{T}D\;d\varepsilon \;d\Omega \;-\int B^{T}C\;dT\;d\Omega$$

To attain the equilibrium state of the entire structure, the equilibrium Equation (2) was assembled as shown in Equation (3).

(3) ∑dF=∑K du−∑dL

where

(4) $$K=\int B^{T}DB\;\;d\Omega$$

(5) $$dL=\int B^{T}C\;dT\;d\Omega$$

In the welding process, no external force acts at the nodes; thus, the increment in the nodal force (dF) is zero, and Equation (3) has a simple form, as shown in Equation (6).

(6) ∑dL=∑K du

To effectively simulate the equations mentioned above, it is crucial to determine the values of K and dL by considering the effect of temperature on the material properties using the relevant mechanical theory. In this study, TEP-FEM was executed using commercial code (ABAQUS 2019) that was self-programmed. Because of its highly nonlinear characteristics, TEP-FEA is typically performed using a sequential coupling method to guarantee convergence.

### 2.2. Numerical Analysis Framework

**Figure 1:**
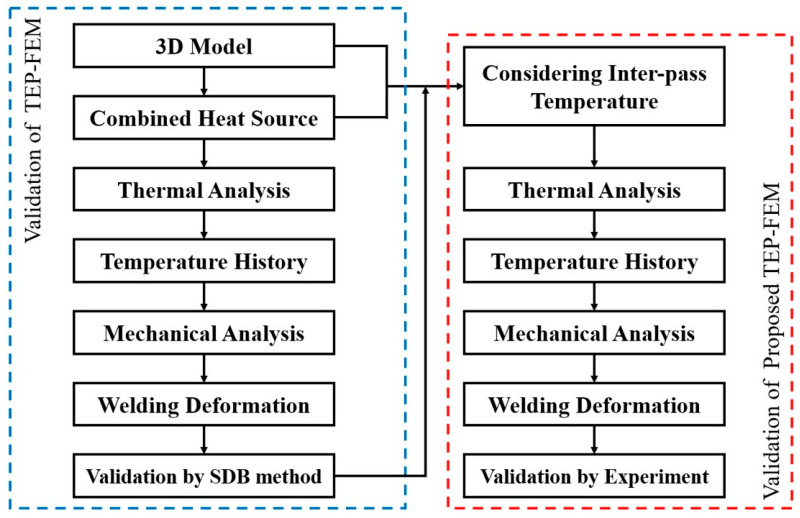
Numerical analysis framework.

A numerical computational framework was used to efficiently analyze the welding deformation, as shown in Figure 1. Welding deformation and residual stress were analyzed using TEP-FEM. First, to validate the accuracy of the TEP-FEM analysis, it was compared with the SDB method, which does not consider the convection and radiation conditions or the temperature history of each bead. Subsequently, considering the interpass temperature, TEP-FEM was performed by increasing the temperature by 100 °C from 200 °C to 1000 °C. Finally, compared with the experiment, the interpass temperature with the lowest welding deformation error was verified.

### 2.3. Structural Parameters of the Welding Plate and Heat Source Model

**Figure 2:**
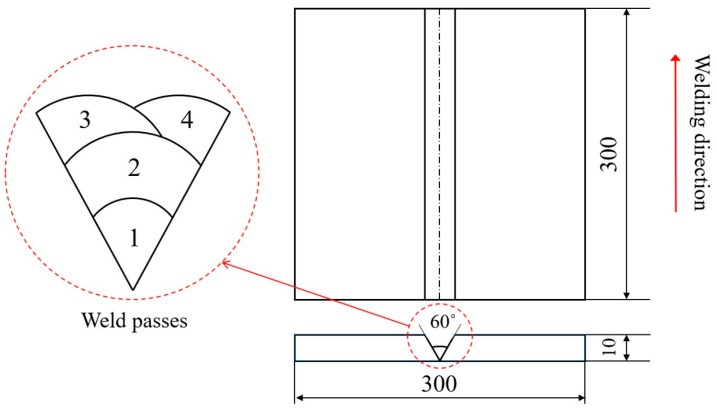
Dimensions of welding plate and arrangements of weld passes.

As shown in Figure 2, a V-groove multi-pass welding plate consisting of four bead layers was fabricated. The base metal was simplified to SUS304 austenitic stainless steel with a thickness of 10 mm. The dimensions of the joints were 300 mm × 300 mm. 

**Figure 3:**
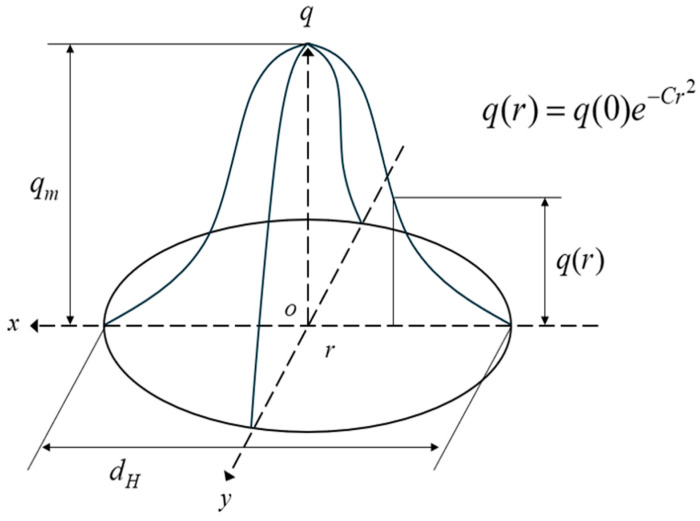
3D Gaussian heat-flux distribution.

**Figure 4:**
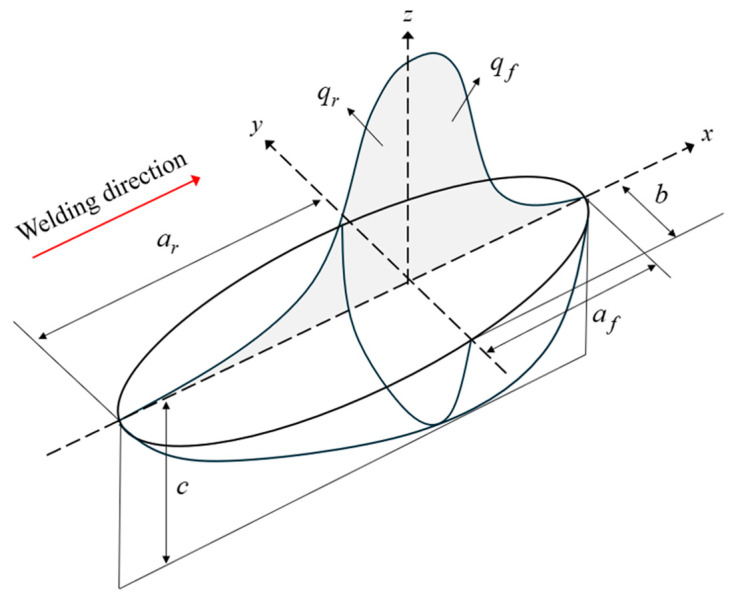
Goldak’s double ellipsoidal heat-flux distribution.

The temperature load used in the thermal analysis was distributed differently depending on the heat source model, and was calculated using the Fourier heat conduction equation. These heat source models include the Gaussian (Figure 3) and double ellipsoidal models (Figure 4) [20]. A 3D Gaussian heat source model is typically utilized to simulate electron beams or laser welding, whereas the Goldak heat source model, characterized by its double ellipsoidal shape, is commonly employed for arc welding. In the latter case, the front and rear ellipsoids can be separately calibrated and fitted to the welding process, thereby enabling the heat source to simulate low-penetration welding. The power density distributions in the front and rear quadrants of Goldak’s double ellipsoidal heat source model used in this study are described using Equations (7) and (8), respectively.

(7) $$q_{f}(x,y,z)=\frac{6\sqrt{3}f_{f}\;\eta \;Q}{a_{f}b\;c\;\pi \sqrt{\pi }}\;\;\exp\;(-\frac{3x^{2}}{a_{f}^{2}}-\frac{3y^{2}}{b^{2}}-\frac{3z^{2}}{c^{2}})$$

(8) $$q_{r}(x,y,z)=\frac{6\sqrt{3}f_{r}\;\eta \;Q}{a_{r}b\;c\;\pi \sqrt{\pi }}\;\;\exp\;(-\frac{3x^{2}}{a_{r}^{2}}-\frac{3y^{2}}{b^{2}}-\frac{3z^{2}}{c^{2}})$$

where $a_{f}$, $a_{r}$, b, and c are the four variables that define the semi-axes for the ellipsoid of the heat source; $a_{f}$ is the front quadrant, $a_{r}$ is the rear quadrant, b is the half width, and c is the depth. Q is the arc heat input (Q=ηIV), where η is the arc efficiency. V and I are the voltage and current of the arc, respectively. The fractions of deposited heat, $f_{f}$ and $f_{r}$, represent the heat apportionments of the heat flux in the front and rear quadrants, respectively, where $f_{f}+f_{r}=2$. The coordinate system for the heat source is defined in Figure 4, where the X-axis is in the direction of the welding motion, Z-axis is in the depth direction, and Y-axis is in the width direction. Other parameters, such as welding speed, welding voltage, and current, are listed in Table 1. The moving heat source was modeled using a user subroutine in ABAQUS.

## 3. TEP-FEM

Unlike the simplified method, TEP-FEM accumulates interpass temperatures during the welding simulation. It also considers convection and radiation, which are not considered in the SDB method, making it difficult to match simulation and experimental results. Therefore, before using TEP-FEM considering the interpass temperature to predict weld deformation, the reliability of TEP-FEM was first validated by comparing the weld deformation with the SDB method when cooled to room temperature.

### 3.1. Thermal Analysis

**Figure 5:**
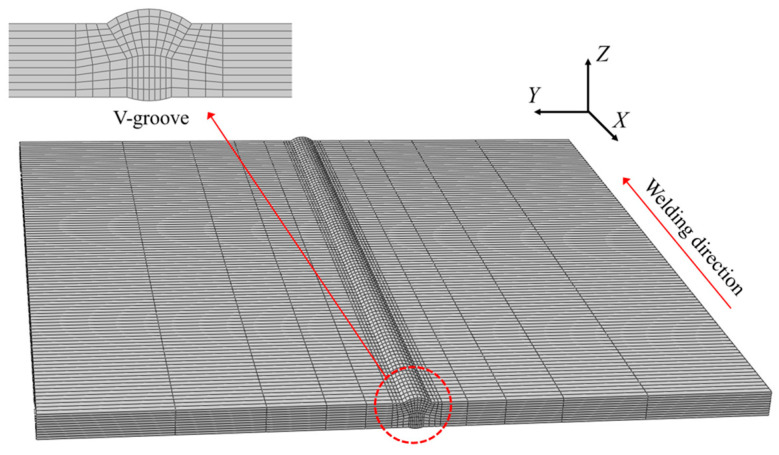
V-groove FE model.

Thermal analysis was conducted using the finite element (FE) model shown in Figure 5, which was designed to match the experimental model in every aspect. The model comprised 24,000 elements and 27,775 nodes. Although the groove shape was simplified to achieve symmetry around the central axis, the overall FE model design was maintained. For the element types, DC3D8 in ABAQUS was used for thermal analysis. During the thermal analysis, the governing equation for the transient heat transfer is shown in Equation (9). The nonlinear isotropic Fourier heat-flux constitutive equations are given in Equation (10).

(9) $$\rho c\frac{\partial T}{\partial t}(x,y,z,t)=-\nabla q(x,y,z,t)+Q(x,y,z,t)$$

**Figure 6:**
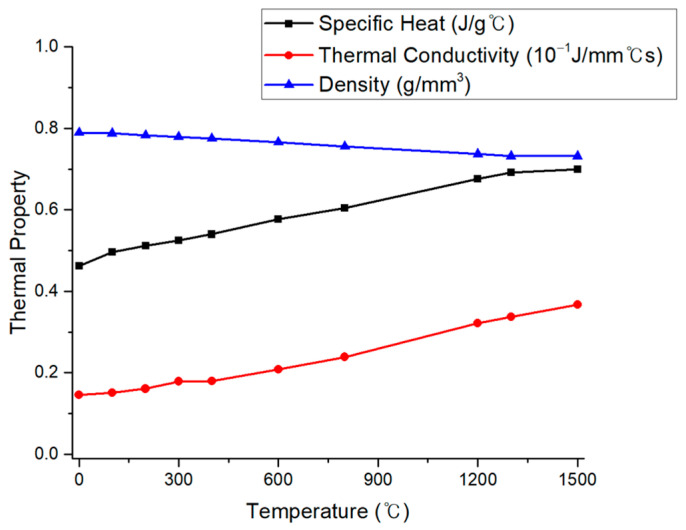
Temperature-dependent thermal properties of SUS304.

(10) q=−k∇T

where ρ is the density, c is specific heat capacity, T is the current temperature, Q is the internal heat generation rate, and k is the thermal conductivity. Thermal properties, such as specific heat and thermal conductivity, change depending on the material used. The thermal properties of SUS304 austenitic stainless steel used in this study are shown in Figure 6 [21]. 

To account for heat losses, thermal radiation and heat transfer on the weld surface were considered. At higher temperatures close to and within the weld zone, radiation losses were more significant, whereas convection losses became more prominent at lower temperatures further away from the weld zone. A user subroutine was created to simulate the combined thermal boundary conditions. The heat transfer coefficient, which depends on temperature, is given by Equation (11) [21].

(11) $$h=\left\{\begin{array}{l} =0.68T\;\times \;10^{-8}\;({W/\mathrm{mm}}^{2})\;\;\;0\;<\;T\;<500\;{}^{\circ}C\\ =(0.231T-82.1)\;\times \;10^{-6}\;({W/\mathrm{mm}}^{2})\;\;\;T\;>\;500\;{}^{\circ}C \end{array}\right\}$$

**Figure 7:**
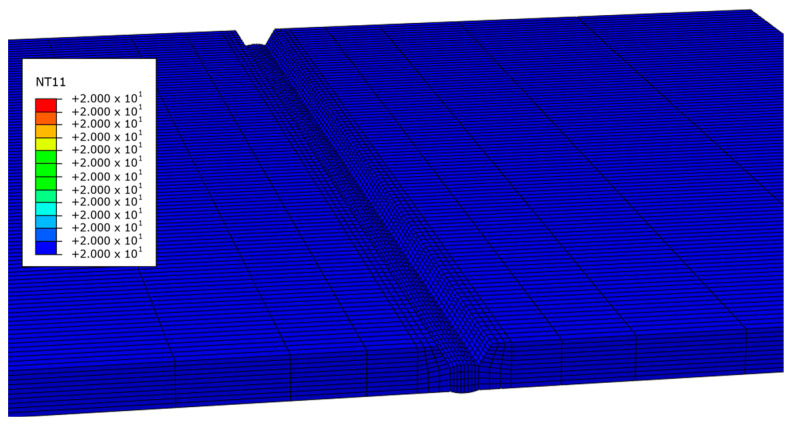
FE model in which the second to fourth beads disappeared using Model Change.

Thermal analysis of the FE model, which consists of four bead layers, does not weld all beads at once but is conducted sequentially using the “Model Change” function in ABAQUS. When the initial bead was welded using Model Change, every component from the first pass was integrated into the FE model, whereas the other passes were left out as shown in Figure 7. 

**Figure 8:**
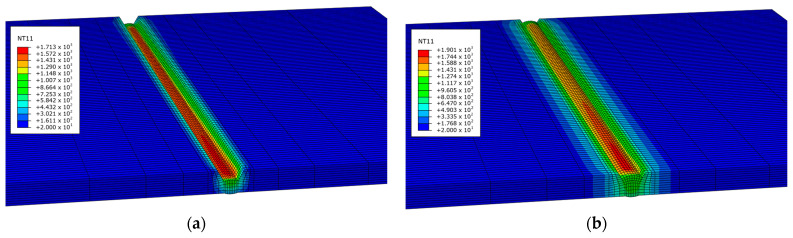
Temperature history of each bead: (**a**) first bead welding; (**b**) second bead welding after regenerate bead using Model Change.

**Figure 9:**
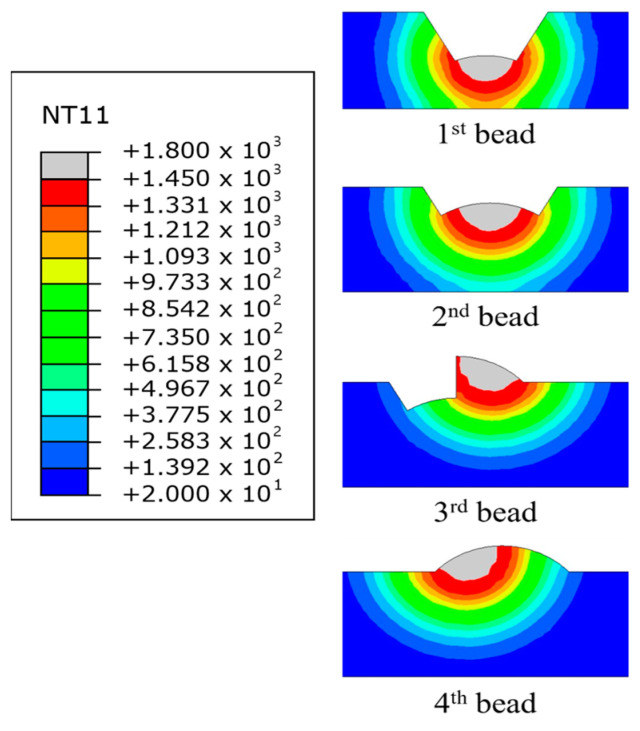
Temperature history of whole V-groove welded joints.

After the first pass, the elements involved in the second weld pass were added to the groove, and the second pass was performed using a moving heat source as shown in Figure 8. The remaining weld passes were completed, and the grooves were fully filled with the weld material. After the welding was completed, the temperature history of the base material was recorded, as shown in Figure 9. This temperature history can be applied to the same FE model for mechanical analysis. 

### 3.2. Mechanical Analysis

**Figure 10:**
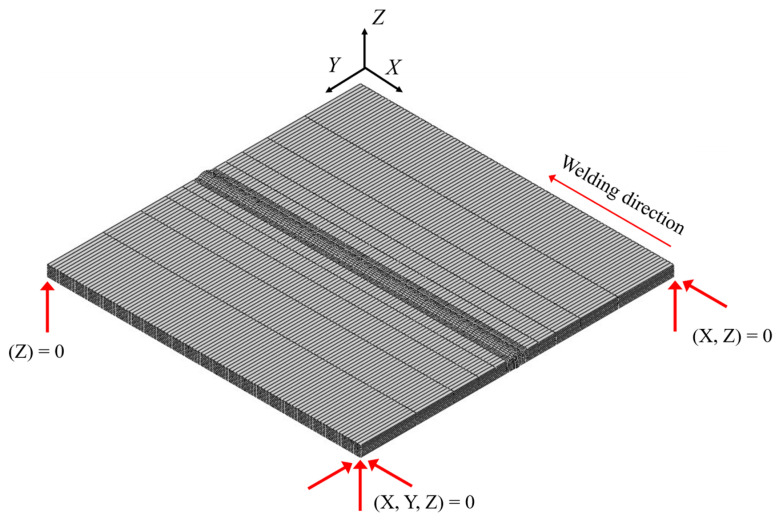
Boundary conditions of FE model.

The same mesh used in the thermal analysis was used for the mechanical analysis, except for the element type (C3D8I in ABAQUS) and boundary conditions. In actual welding, there are no boundary conditions. However, in the mechanical analysis, boundary conditions are provided, as shown in Figure 10, to prevent rigid-body modules. 

**Figure 11:**
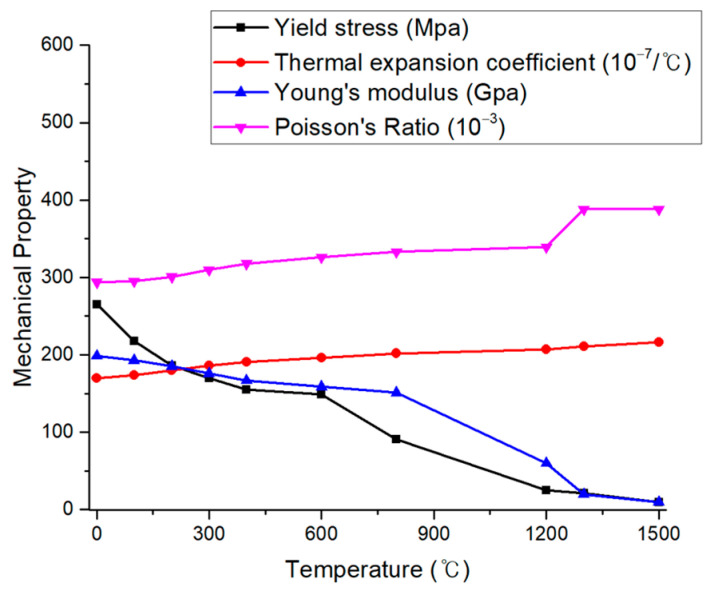
Temperature-dependent mechanical properties of SUS304.

A mechanical analysis was conducted using the temperature history computed by the thermal analysis as the thermal load. In this study, we did not consider the solid-state phase transformation of the weld base material and molten metal. Additionally, because the creep strain is negligible, the total strain of the element can be calculated as the sum of the three strains, as expressed in Equation (12).

(12) $$\{d\varepsilon \}\;\;=\;\;\{d\varepsilon^{e}\}\;\;+\;\;\{d\varepsilon^{p}\}\;\;+\;\;\{d\varepsilon^{th}\}\;\;$$

where the elastic strain is represented by $d\varepsilon^{e}$, plastic strain by $d\varepsilon^{p}$, and thermal strain by $d\varepsilon^{th}$. During the mechanical analysis, isotropic Hooke’s law was utilized to model the elastic strain, with Young’s modulus and Poisson’s ratio being temperature-dependent, as shown in Figure 11 [21].

**Figure 12:**
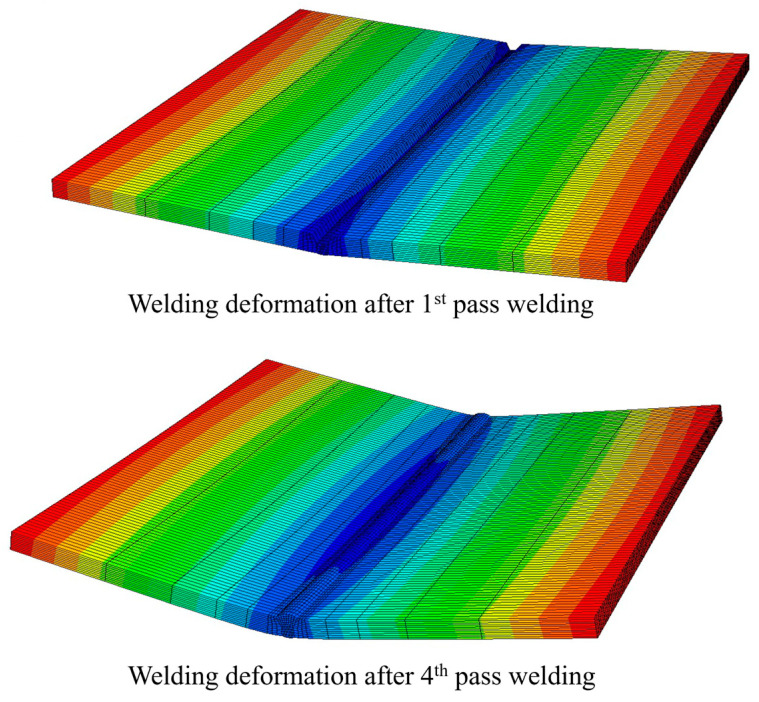
Multi-pass welding deformation of the FE model.

To represent the plastic strain that changes with temperature, the Von Mises yield, temperature-dependent mechanical properties, and isotropic strain-hardening models were used. As welding progressed, the weld plate deformed into a V-shape. However, in multi-pass welding, this deformation accumulates; therefore, the more welding beads pile up, the greater the amount of deformation, as shown in Figure 12. 

Therefore, the total amount of angular distortion that occurred in the z-direction was equal to the sum of the amount of deformation that occurred in each pass, as shown in Equation (13).

(13) $$dz^{total}\;\;=\;\;dz^{1st}+dz^{2nd}+dz^{3rd}+dz^{4th}\;\;$$

**Figure 13:**
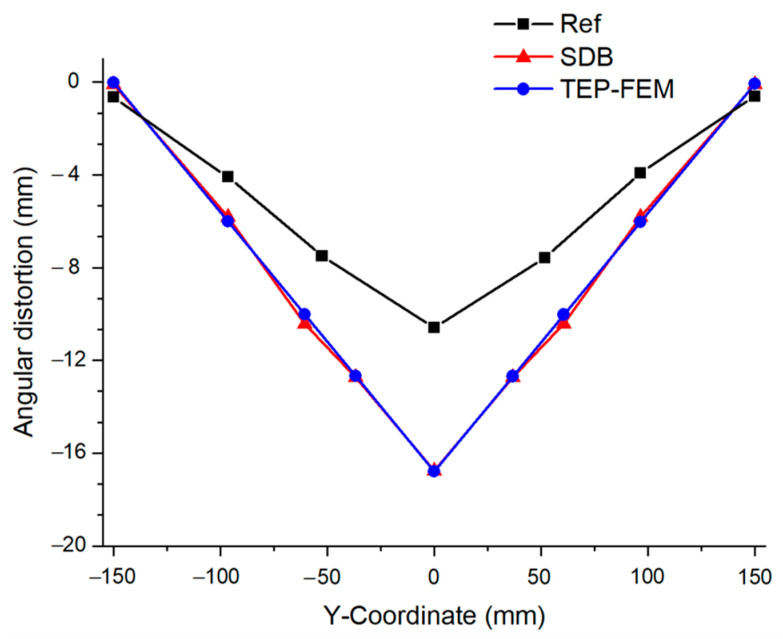
Multi-pass welding deformation cooled to room temperature.

However, as the welding is assumed to cool to room temperature in TEP-FEM, such as SDB, it derives values that differ from the actual welding deformation [22], as shown in Figure 13. However, the error was very small compared with the SDB result [19].

## 4. TEP-FEM Considering Interpass Temperature

**Figure 14:**
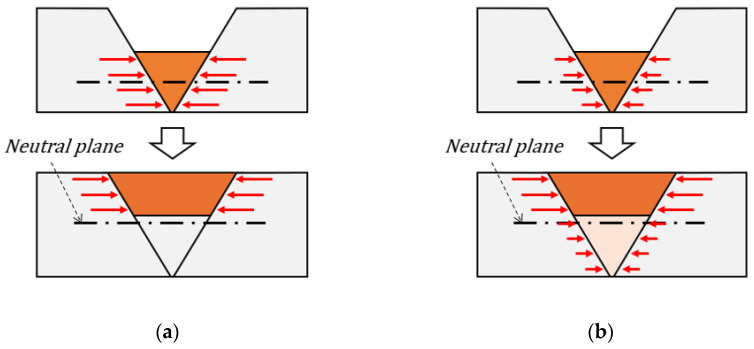
Internal shrinkage in multi-pass welding: (**a**) when an interpass welded area is cooled to room temperature; (**b**) when an interpass welded area is not cooled to room temperature.

The same method and mesh were used as previously described for the TEP-FEM; however, in this section, weld deformation is analyzed considering the interpass temperature. In this case, as mentioned in Section 3, solid-state phase transformation was not considered; however, an alternative to phase transformation can be provided by considering the shrinkage by volume of the weld zone according to the interpass temperature. Figure 14 shows the internal shrinkage in the heated area of a multi-pass weld. A weld cooled to room temperature may generate internal shrinkage in the heated area, as shown in Figure 14a. In addition, each bead shrank without reducing the overall bending moment. For this reason, more welding deformation may occur if the interpass temperature is not considered. However, as shown in Figure 14b, a weld that is not cooled to room temperature will merge with other beads without completing internal shrinkage. Therefore, the heated area where the internal shrinkage occurred became thicker, and the overall bending moment decreased.

### 4.1. Thermal Analysis

**Figure 15:**
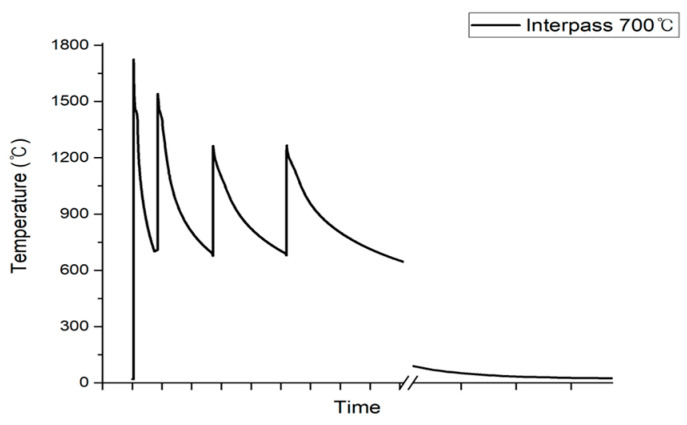
Temperature of the first bead over time considering interpass.

Thermal analysis considering the interpass temperature was performed in 100 °C increments from 200 °C to 1000 °C. An interpass temperature of 100 °C was excluded because there was no notable difference compared to the interpass room temperature (20 °C). Welding was performed continuously at a specific temperature without completely cooling the beads to room temperature. The fourth bead welding step, which was the final step of this model, was completed, and the entire bead was cooled to room temperature. The higher the interpass temperature, the more heat is accumulated during multi-pass welding, thereby increasing the time required to reach the target temperature, as shown in Figure 15.

Additionally, as the number of passes increased, the time gradually increased. However, even when this time was increased, the overall welding process time was ultimately shorter in all cases than when the interpass temperature was not considered. 

**Figure 16:**
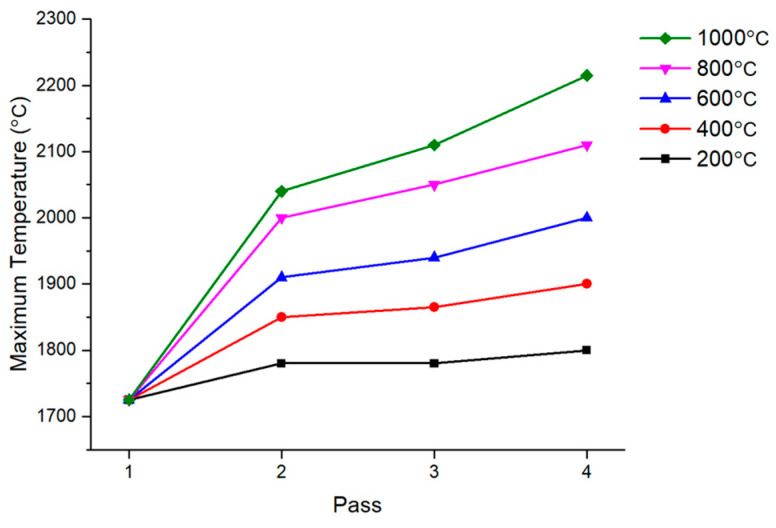
Maximum temperature by interpass temperature.

**Figure 17:**
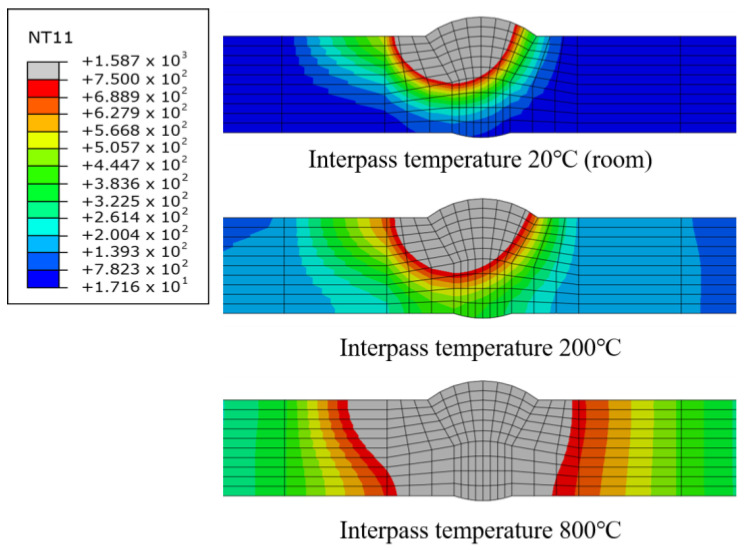
The temperature history during multi-pass welding.

As the interpass temperature increased, the maximum temperature observed in the temperature history also increased, as shown in Figure 16. When welding was performed while cooling to room temperature, the maximum temperature was 1725 °C. However, at an interpass temperature of 1000 °C, the maximum temperature increased to 2215 °C. Additionally, the increased maximum temperature directly affected the HAZ. Figure 17 shows the temperature history during multi-pass welding when the temperature was the highest. As indicated in the legend, 750 °C is the temperature at which martensite, having a negative effect on mechanical properties, mainly occurs.

Comparing the interpass temperatures of 20 °C and 200 °C, the areas exceeding 750 °C appear similar. However, at 200 °C, the temperature increased slightly in the area around the HAZ. Comparing the internal temperatures of 200 °C and 800 °C, we can see that the HAZ clearly expanded at 800 °C. As the HAZ changes rapidly depending on the interpass temperature, the TEP-FEM proposed in this study should be used instead of the previously used SDB study. However, although an increase in the HAZ reduces welding deformation, it may also have a negative effect on mechanical properties later, so the pass temperature must be appropriately controlled.

### 4.2. Mechanical Analysis

**Figure 18:**
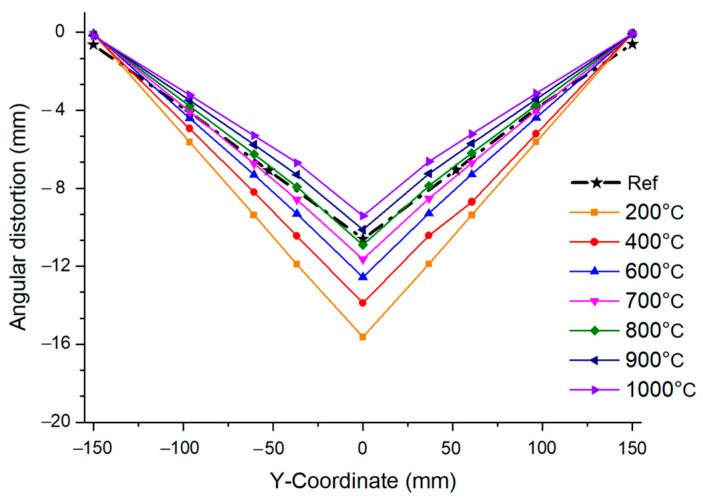
Angular distortion by interpass temperature.

Similar to the previous analysis, a mechanical analysis considering the interpass temperature was performed using the same mesh, and the total amount of angular distortion was calculated using Equation (13). As the interpass temperature increased from 200 °C to 1000 °C, the angular distortion decreased inversely. However, if the interpass temperature exceeded 800 °C, the angular distortion value decreased further than the experimental value [22] and the error increased. Therefore, the temperature that best predicts welding deformation must be appropriately controlled. Figure 18 shows the total welding deformation using TEP-FEM according to the interpass temperature.

**Figure 19:**
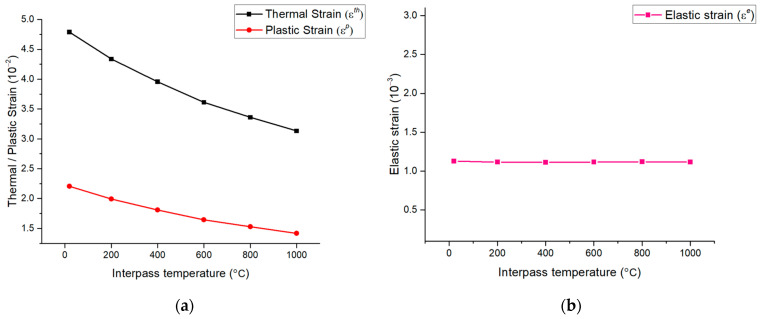
Strain by interpass temperature: (**a**) thermal and plastic strains; (**b**) elastic strain.

When the interpass temperature was 800 °C, the angular distortion was 10.9 mm. The deformation error was 2.62% compared to the actual welding angular distortion [22]. However, when the interpass temperature was 1000 °C, the angular distortion was the lowest at 9.4 mm, but the error increased by 11.23%. In other words, as the interpass temperature increases, decreasing the angular distortion implies that the total strain expressed in Equation (13) decreases. Figure 19 shows the various strains depending on the interpass temperature. Among the total strains, the thermal, plastic, and elastic strains accounted for the largest proportion, in that order. The thermal strain was more than twice as large as the plastic strain, as shown in Figure 19a.

**Figure 20:**
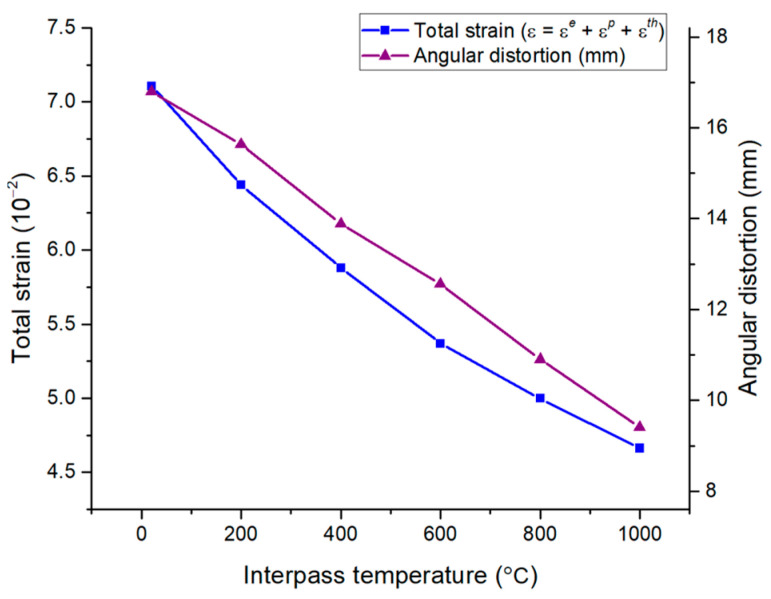
Total strain and angular distortion by interpass temperature.

However, the elastic strain was relatively small compared to the thermal and plastic strains, as shown in Figure 19b. Additionally, the elastic strain was almost unaffected by the interpass temperature, and its value also remained constant. As a result, because the total strain was obtained by adding all the thermal, plastic, and elastic strains, it was confirmed that the total strain decreased as the interpass temperature increased. Additionally, when comparing the total strain and angular distortion together, it was confirmed that the trends were consistent, as shown in Figure 20.

**Figure 21:**
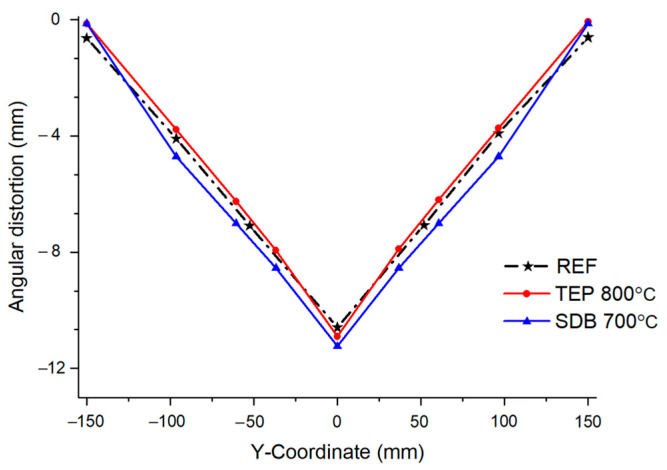
Angular distortion comparison between SDB and TEP-FEM.

Finally, the angular distortion of the TEP-FEM and the SDB used in previous studies [19] was compared to verify the accuracy of the TEP-FEM in predicting welding deformation. When the interpass temperature was 700 °C, the angular distortion of SDB was 11.24 mm. However, the angular distortion of TEP-FEM was 10.9mm. Furthermore, the deformation prediction error of TEP-FEM was 3.62% lower than that of SDB, as shown in Figure 21.

## 5. Effect of Welding Parameters on TEP-FEM

Through the above welding deformation analysis using TEP-FEM, we confirmed that as the interpass temperature increased, the angular distortion decreased. Additionally, the deformation prediction error was the lowest when the interpass temperature was 800 °C. However, the optimal interpass temperature for all welds is not 800 °C. Welding deformation prediction is complex, and many factors other than the interpass temperature affect welding deformation. Therefore, to improve the rationality of welding deformation prediction considering the interpass temperature, another welding deformation analysis was performed in which the interpass temperature (800 °C) was the same, but the welding parameters, such as the welding speed and welding current, were changed.

### 5.1. Welding Speed

Welding speed is one of the important parameters in performing welding. Before the development of robotic welding, the welding speed could only be controlled by the welder, and inconsistent welding speed had a negative impact on welding quality. Additionally, even if the same heat source is used, slowing down the welding speed results in greater thermal distortion because the heat source remains in the weld structure longer. Therefore, in this section, we analyze the effect of changing the welding speed on angular distortion by using the same heat source and interpass temperature. The detailed welding parameters are specified in Table 2.

The thermal and mechanical analyses performed in this section also used the same FE model (Figure 5 and Figure 10) and TEP-FEM considering the interpass temperature.

**Figure 22:**
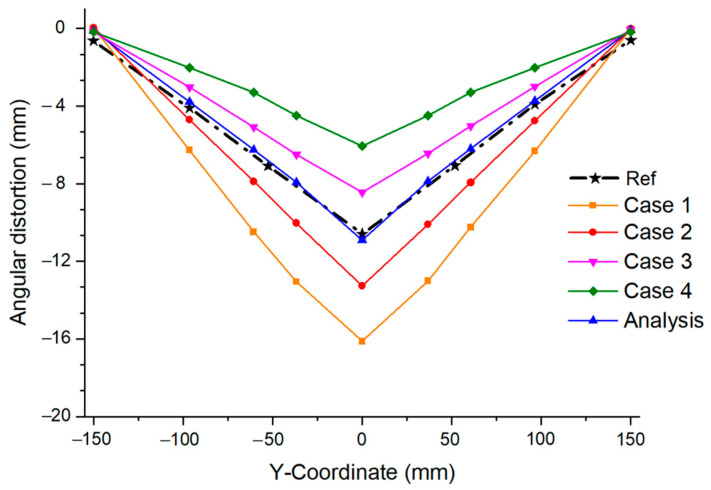
Angular distortion by welding speed.

Even though the same heat source and interpass temperature were used, the angular distortion that changed rapidly depending on the welding speed can be confirmed, as shown in Figure 22. In addition, the range of variation in the angular distortion based on the welding speed was wider than that based on the interpass temperature. Therefore, considering these results, it becomes easier to predict welding deformation if the welder first determines the welding speed rather than the interpass temperature.

### 5.2. Welding Current

The welding current is not a parameter that the welder modifies as much as the welding speed, but it still has a significant impact on weld deformation. Additionally, the welding current has a direct impact on heat source modeling, determining whether the net heat input increases or decreases. If the welding current is too low, the arc becomes unstable, and the penetration depth decreases owing to a decrease in the net heat input. This can cause defects, such as slag in the weld, or reduce productivity. Conversely, if the welding current is too high, defects such as undercuts occur in the weld area. Additionally, if the heat penetration depth becomes too large owing to an increase in the net heat input, unexpected excessive deformation occurs. Owing to these various defects, the welding current is preselected within an appropriate range before operation.

Angular distortion analysis due to changes in welding current was confirmed using the same heat source and interpass temperature as the analysis of changing the welding speed. The detailed welding parameters are specified in Table 3.

**Figure 23:**
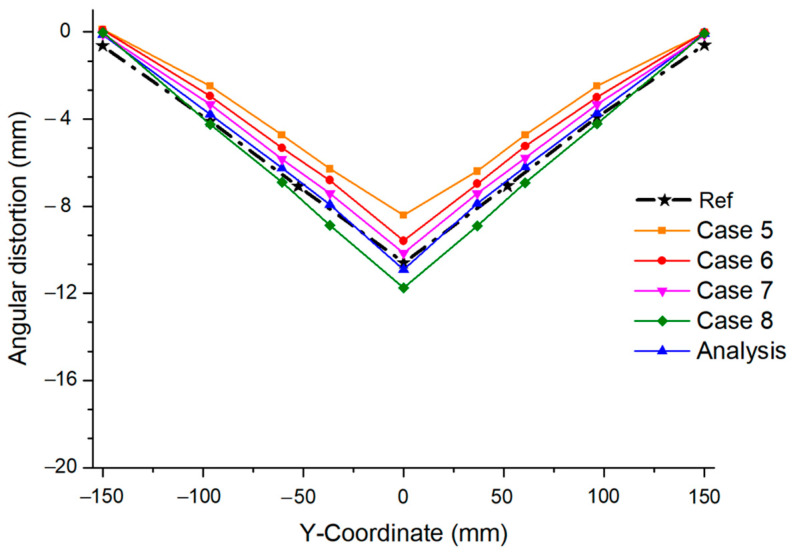
Angular distortion by welding current.

Figure 23 shows the angular distortion caused by the welding current. Compared to the welding speed, the fluctuation range of the angular distortion owing to changes in the welding current is small. Therefore, if we determine the welding speed before the welding current, it may be easier to determine the interpass temperature at which the error in the predicted deformation is minimal. However, the welding speed and current cannot be modified midway during the welding. Conversely, the interpass temperature can be modified at any time depending on the work environment because it takes sufficient time through convection and radiation to reach the target interpass temperature. Additionally, the interpass temperature setting is more flexible than the other parameters because different temperatures can be specified for each bead when performing multi-pass welding.

## 6. Conclusions

In this study, we proposed a TEP-FEM that considers the interpass temperature to predict deformation in multi-pass welding. This method is based on the principle that the interpass temperature is not cooled to room temperature during welding to ensure the overall process efficiency. Therefore, in this study, the interpass temperature was increased from 200 °C to 1000 °C, and thermal and mechanical analyses were performed. Consequently, the error caused by the assumption that the beads cooled to room temperature was substantially minimized. Additionally, the temperature that most accurately predicted the actual welding deformation (10.59 mm) was 800 °C, with a predicted deformation of 10.9 mm. Additionally, this method yields more beneficial results than the SDB approach employed in previous studies [19]. The results are summarized as follows:

(1) The SDB method is a simple analysis method that can only check the deformation results, whereas TEP-FEM can simultaneously check the deformation results, accumulated temperature history, and total time to weld.
(2) TEP-FEM confirms that the overall process time decreases as the interpass temperature changes.
(3) The deformation error of TEP-FEM was 2.4%, which is markedly lower than the 6.14% error reported in a previous study (SDB 700 °C).
(4) It was confirmed that the thermal strain and plastic strain decreased in the same way as the angular distortion when the interpass temperature increased.
(5) The elastic strain was not affected by the interpass temperature, unlike the thermal and plastic strains.

However, TEP-FEM considering the interpass temperature also has disadvantages. If the interpass temperature is excessively raised to reduce welding deformation, the deformation prediction error may increase. Additionally, as the interpass temperature increases, the maximum temperature also increases, which can change the size of the heat-affected zone that is critical to welding. Finally, in this study, another analysis was performed by changing various welding parameters to increase the rationality of the welding deformation prediction considering the interpass temperature. As a result, it was confirmed that to minimize the error in the predicted deformation by changing the interpass temperature, the welding parameters (welding speed first, welding current second) must first be determined rather than the interpass temperature.

## Figures and Tables

**Table 1:** Welding parameters used in multi-pass welding.

Parameter	Value
Fractions of front heat ($f_{f}$)	1.4
Fractions of front rear ($f_{r}$)	0.6
Welding voltage (V)	21
Welding current (I)	205
Arc efficiency (η)	0.7
Welding speed (mm/min)	180

**Table 2:** Welding parameters configured at different welding speeds.

Parameter	Analysis	Case 1	Case 2	Case 3	Case 4
Fractions of front heat ($f_{f}$)	1.4	1.4	1.4	1.4	1.4
Fractions of front rear ($f_{r}$)	0.6	0.6	0.6	0.6	0.6
Welding voltage (V)	21	21	21	21	21
Welding current (I)	205	205	205	205	205
Arc efficiency (η)	0.7	0.7	0.7	0.7	0.7
Welding speed (mm/min)	180	120	150	210	240
Interpass temperature (°C)	800	800	800	800	800

**Table 3:** Welding parameters configured at different welding currents.

Parameter	Analysis	Case 5	Case 6	Case 7	Case 8
Fractions of front heat ($f_{f}$)	1.4	1.4	1.4	1.4	1.4
Fractions of front rear ($f_{r}$)	0.6	0.6	0.6	0.6	0.6
Welding voltage (V)	21	21	21	21	21
Welding current (I)	205	175	185	195	215
Arc efficiency (η)	0.7	0.7	0.7	0.7	0.7
Welding speed (mm/min)	180	180	180	180	180
Interpass temperature (°C)	800	800	800	800	800

## Data Availability

The data presented in this study are available on request from the corresponding author.
